# Synthesis and structure of 4-[(2,3,4,5,6-pentafluoro­phen­oxy)carbon­yl]phenyl 4-(dodec­yloxy)benzoate

**DOI:** 10.1107/S2056989026003920

**Published:** 2026-04-17

**Authors:** Khaleel Ahmed, B. Bommalingaiah, G. N. Venkatareddy, H. T. Srinivasa, H. C. Devarajegowda, B. S. Palakshamurthy

**Affiliations:** a Department of Physics Yuvaraja's College University of Mysore,Mysore Karnataka-570005 India; b Department of Physics, Government Science College, Chitradurga, Karnataka-577501, India; c Raman Research Institute, C V Raman Avenue Sadashivanagar Bengaluru Karnataka-560086 India; d Department of PG Studies and Research in Physics UCS Tumkur University, Tumkur Karnataka-572103 India; University of Aberdeen, United Kingdom

**Keywords:** crystal structure, dodec­yloxy, perfluoro­phen­oxy, Hirshfeld surface, hydrogen bond

## Abstract

In the title compound, C_32_H_33_F_5_O_5_, the dihedral angle between the central carbonyl­pheyl and peripheral perfluoro­phen­oxy and (dodec­yloxy)benzoate rings are 85.24 (2) and 78.98 (2)^ο^, respectively, indicating that the central ring is almost normal to both adjacent rings. The pendant C_12_ alkyl chain adopts an all-*anti* conformation. In the crystal, weak C—H⋯O hydrogen bonds connect the mol­ecules, forming *S*(7) chains propagating along the [010] direction. The packing is consolidated by C—F⋯π inter­actions and weak π–π stacking. The Hirshfeld surface analysis reveals that the major contributions to the two-dimensional fingerprint plots are from H⋯H (45%),F⋯H/H⋯F (18.5%), O⋯H/H⋯O (9.7%), C⋯H/H⋯C (9.4%), F⋯C/C⋯F (7.3%), and F⋯F (1.9%) contacts. An energy framework calculation shows that dispersion energy (–383.4 kJ mol^−1^) makes by far the largest contribution.

## Abstract 2

The title mol­ecule was synthesized by an acid–phenol coupling reaction. A Hirshfeld surface analysis was performed to qu­antify the inter­molecular inter­actions in the crystal.

## 1. Chemical context

Phenyl­benzoate-based three-ring calamitic liquid crystals incorporating a 4-(dodec­yloxy)benzoate terminal unit are well-established mesogens in which the dodec­yloxy chain promotes layered organization, while ester linkages preserve the required rod-like geometry (Cakar *et al.*, 2022 ▸). The introduction of a perfluoro­phen­oxy group at the opposite terminus is expected to influence both inter­molecular inter­actions and physicochemical properties through fluorination (Ashmawy *et al.*, 2017 ▸; Podruczna *et al.*, 2014 ▸). Beyond their mesomorphic behaviour, derivatives bearing the 4-(dodec­yloxy)benzoate motif have attracted attention due to their biological activities. In closely related systems, structural modification of the terminal substituent and alkyl chain length has been shown to significantly affect biological performance. For instance, bis­(dodec­yloxy)benzoate–poly(amido­amine) conjugates exhibit pronounced anti­cancer activity against a range of human cancer cell lines (Castillo-Rodrez *et al.*, 2023 ▸), while flutamide-linked 3,5-bis­(dodec­yloxy)benzoate derivatives demonstrate effective inhibition toward U-251, PC-3, K-562 and HCT-15 cell lines (Medina-Rojas *et al.*, 2020 ▸; Lukáč *et al.*, 2024 ▸). Similarly, incorporation of long alkyl chains in heterocyclic benzoate derivatives enhances corrosion inhibition efficiency, indicating strong surface adsorption driven by hydro­phobic inter­actions (Kadhim *et al.*, 2023 ▸).

More generally, elongation of alkyl chains in organic mol­ecules is known to enhance lipophilicity, thereby improving membrane permeability and facilitating cellular uptake, which is a key factor in drug design. This effect has been demonstrated in several systems, including alkyl­ated caffeic acid derivatives exhibiting anti­cancer properties, cinnamic acid analogues showing anti-tuberculosis activity (De *et al.*, 2011 ▸), and amide-based compounds with improved anti-inflammatory behaviour upon chain extension (Matta *et al.*, 2020 ▸). In this context, the present structural study of the title compound, C_32_H_33_F_5_O_5_ (**I**), provides insight into the mol­ecular conformation of the C_12_-alkyl chain and the inter­molecular contacts governing crystal packing, which may contribute to both its mesomorphic characteristics and potential biological inter­actions (Koshti *et al.*, 2023 ▸; Singh *et al.*, 2016 ▸).
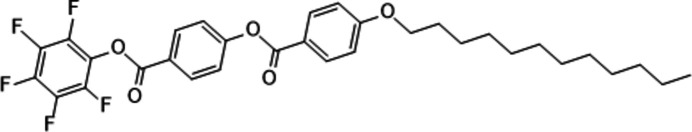


## 2. Structural commentary

**Figure 1:**
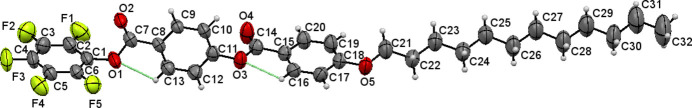
The mol­ecular structure of (**I**) showing 50% probability ellipsoids. Short intramolecular —H⋯O contacts are shown as green dashed lines.

The mol­ecular structure of (**I**) is shown in Fig. 1 ▸. The dihedral angle between the aromatic rings of the perfluoro­phen­oxy (C1–C6) and carbonyl­phenyl (C8–C13) fragments are 85.24 (2)° and the corresponding dihedral angle for the carbonyl­phenyl and (dodec­yloxy)benzoate (C15–C20) rings is 78.98 (2)°, thus, the central ring is close to normal to both peripheral rings. The torsion angles associated with the C8—C7—O1—C1 and C15—C14—O3—C11 ester linkages are 175.6 (3) and 172.2 (3)°, respectively, indicating the expected *anti*-periplanar conformations. The pendant C_12_ alkyl chain adopts an all-*anti* conformation with the largest torsion angle deviation from ±180° being −173.1 (4)° for C21—C22—C23—C24. Two short intra­molecular C—H⋯O contacts (Table 1 ▸) are observed. Otherwise, the bond length and the bond angles may be regarded as normal.

.

## 3. Supra­molecular features

**Figure 2:**
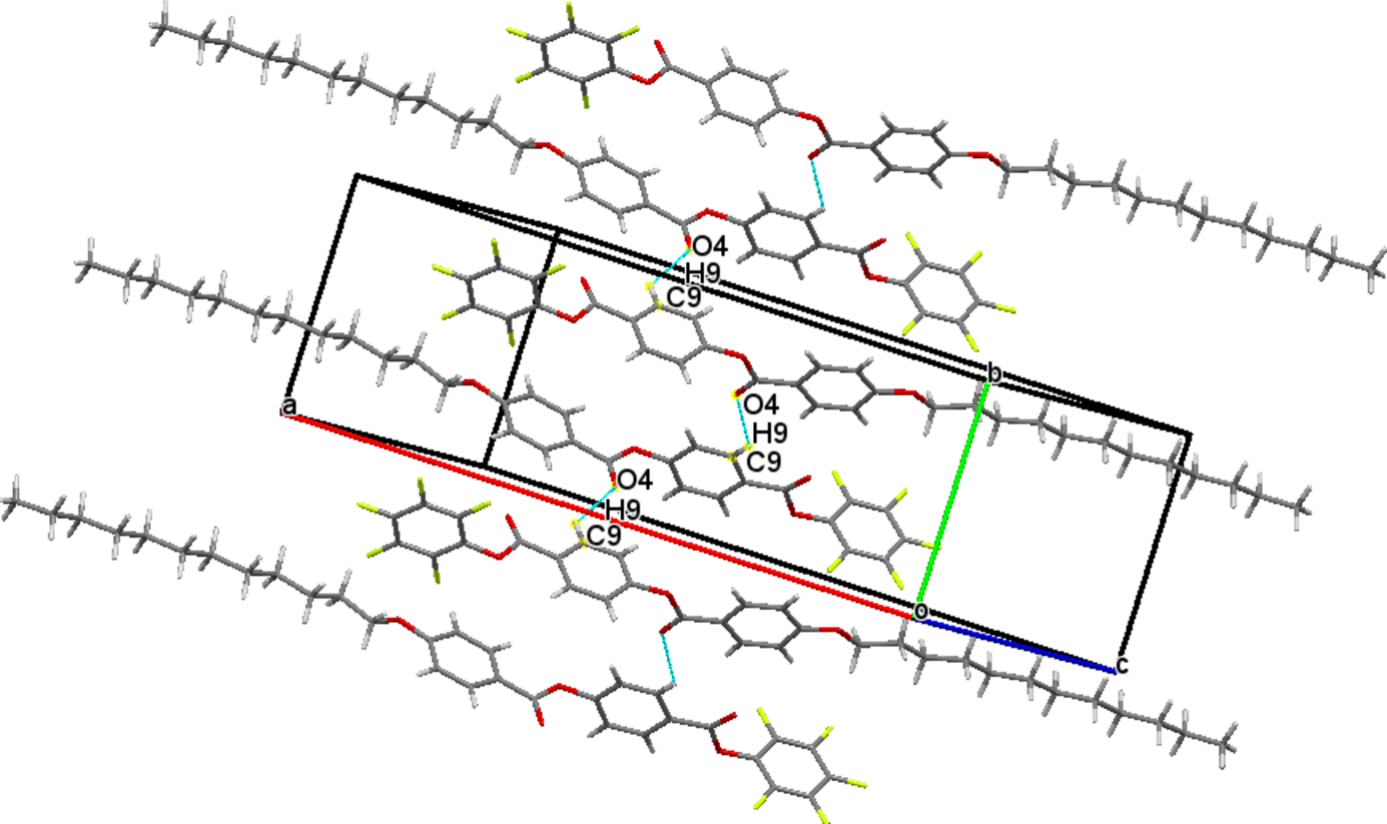
Detail of the packing of (**I**) showing C—H⋯O hydrogen bonds (blue dashed lines) connecting the mol­ecules into *S*(7) [010] chains.

**Figure 3:**
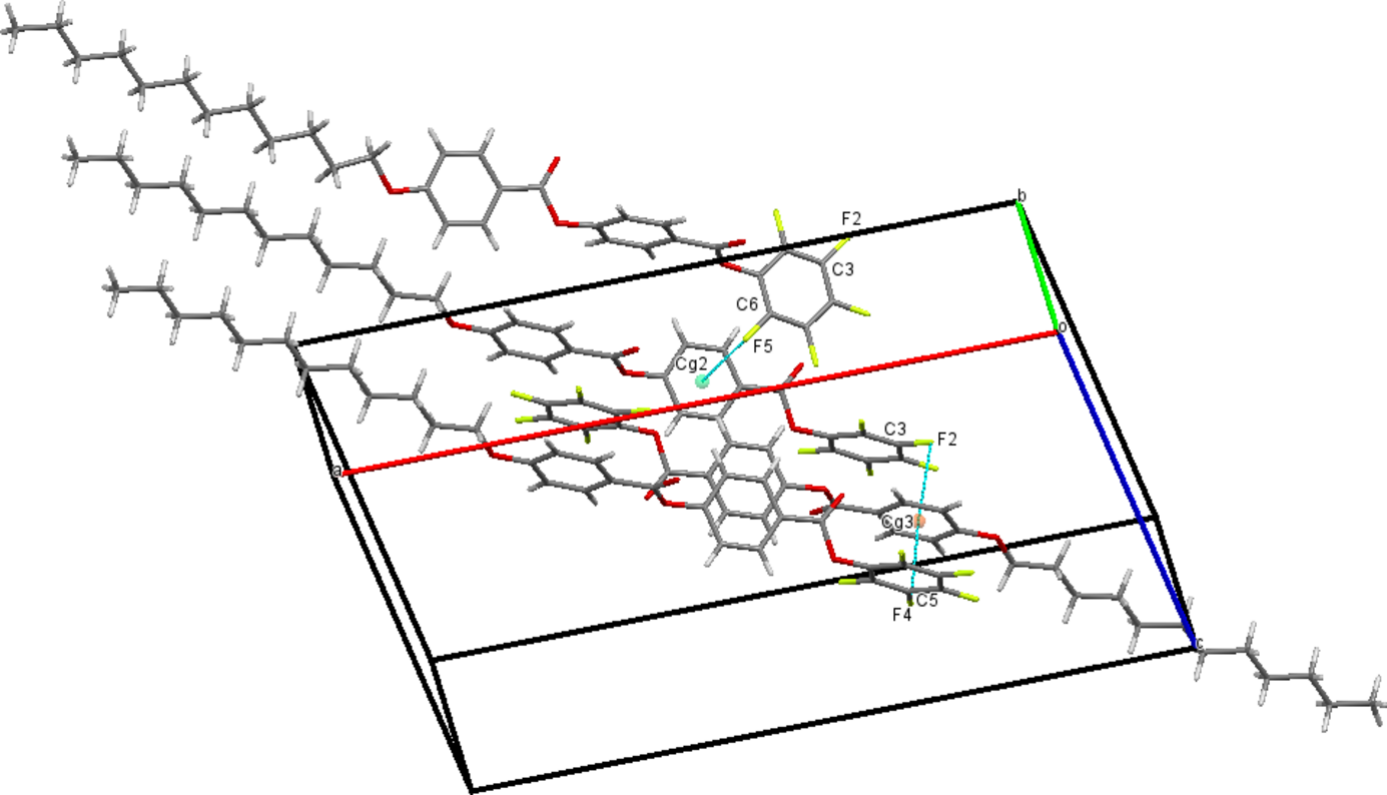
Detail of the packing of (**I**) showing C—F⋯π inter­actions as blue dashed lines.

**Figure 4:**
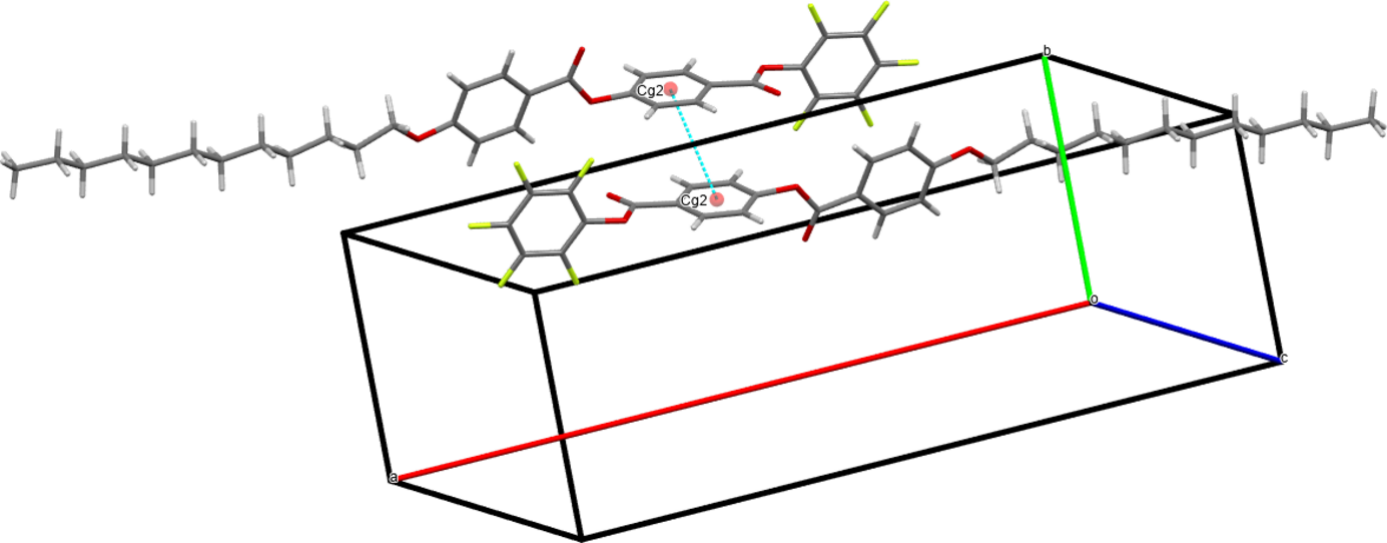
Detail of the packing of (**I**) showing aromatic π–π stacking.

In the crystal, weak C9—H9⋯O4 hydrogen bonds (Table 1 ▸) connect the mol­ecules into infinite *S*(7) chains propagating along the [010] direction as shown in Fig. 2 ▸. The packing is consolidated by C—F⋯π inter­actions (Fig. 3 ▸, Table 1 ▸) , *viz*.: C6—F5⋯*Cg*2, C3—F2⋯*Cg*3 and C5—F4⋯*Cg*3, where *Cg*2 and *Cg*3 are the centroids of the C8–C13 and C15–C20 rings, respectively. Very weak aromatic π–π stacking between pairs of *Cg*2 rings related by inversion symmetry with a centroid–centroid distance of 4.079 (2) Å and a slippage of 2.212 Å is also seen (Fig. 4 ▸).

## 4. Hirshfeld surface analysis

**Figure 5:**
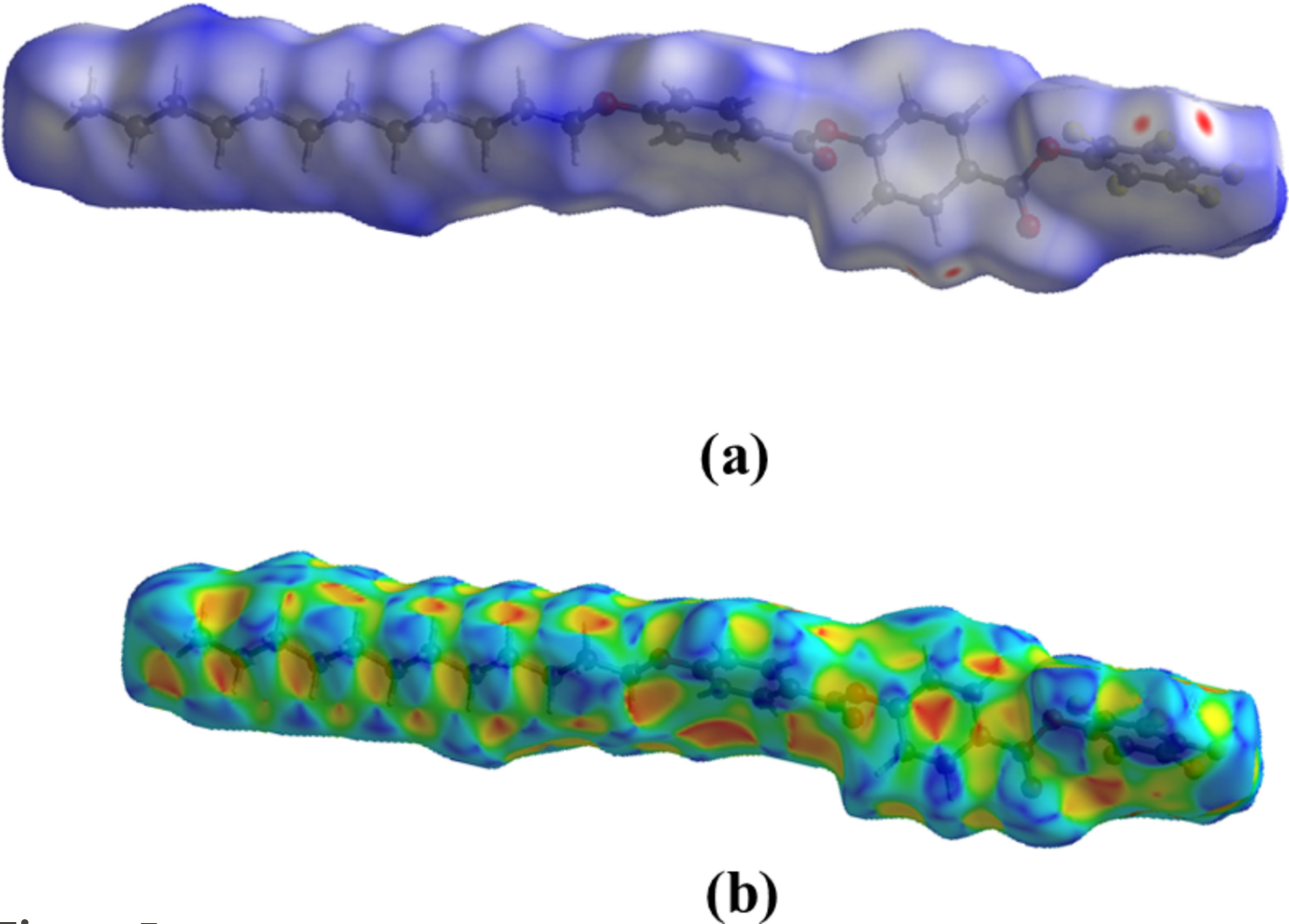
View of the three-dimensional Hirshfeld surface of (**I**) plotted over (*a*) *d*_norm_ and (*b*) shape-index.

**Figure 6:**
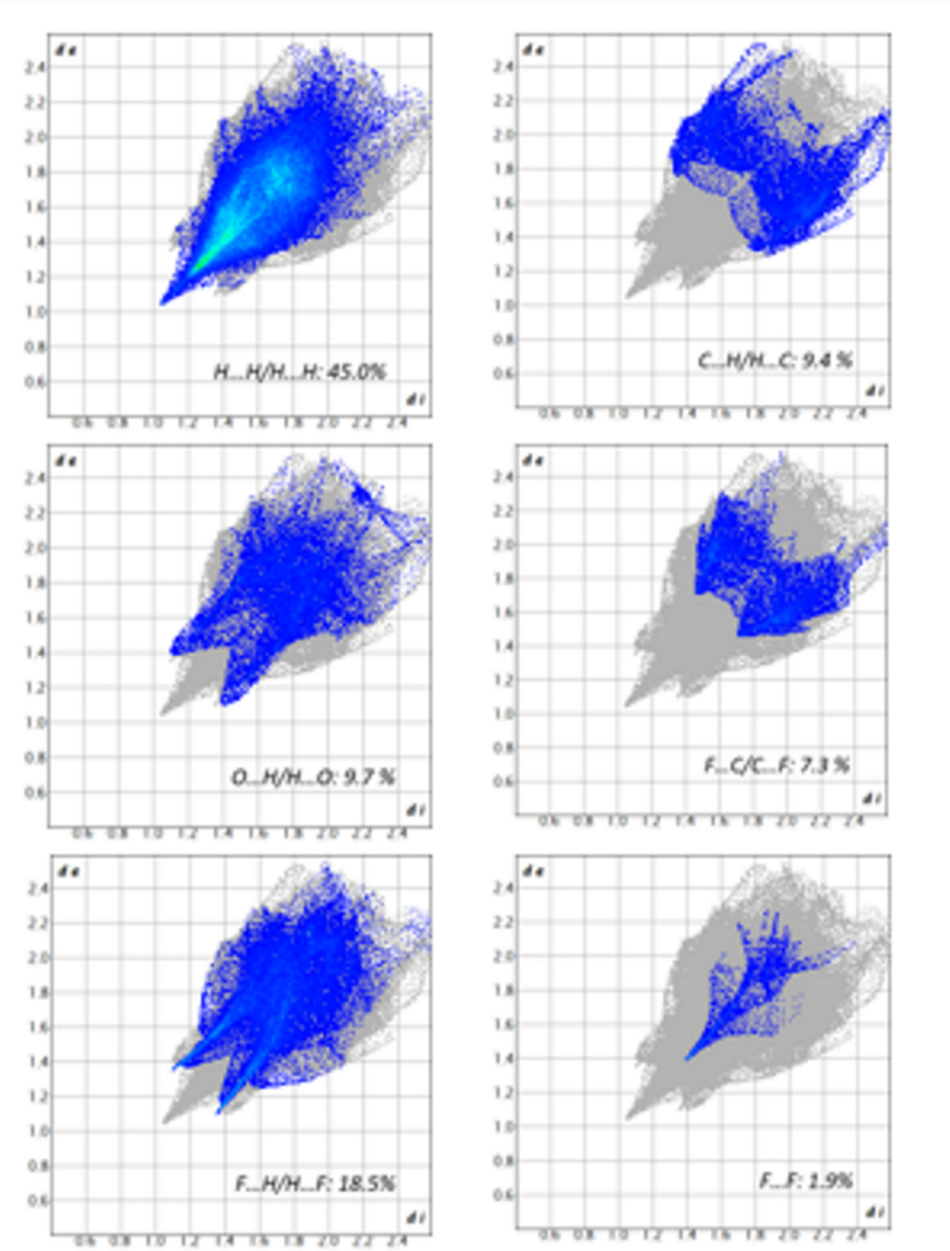
The two-dimensional fingerprint plots for (**I**) for different contact types.

**Figure 7:**
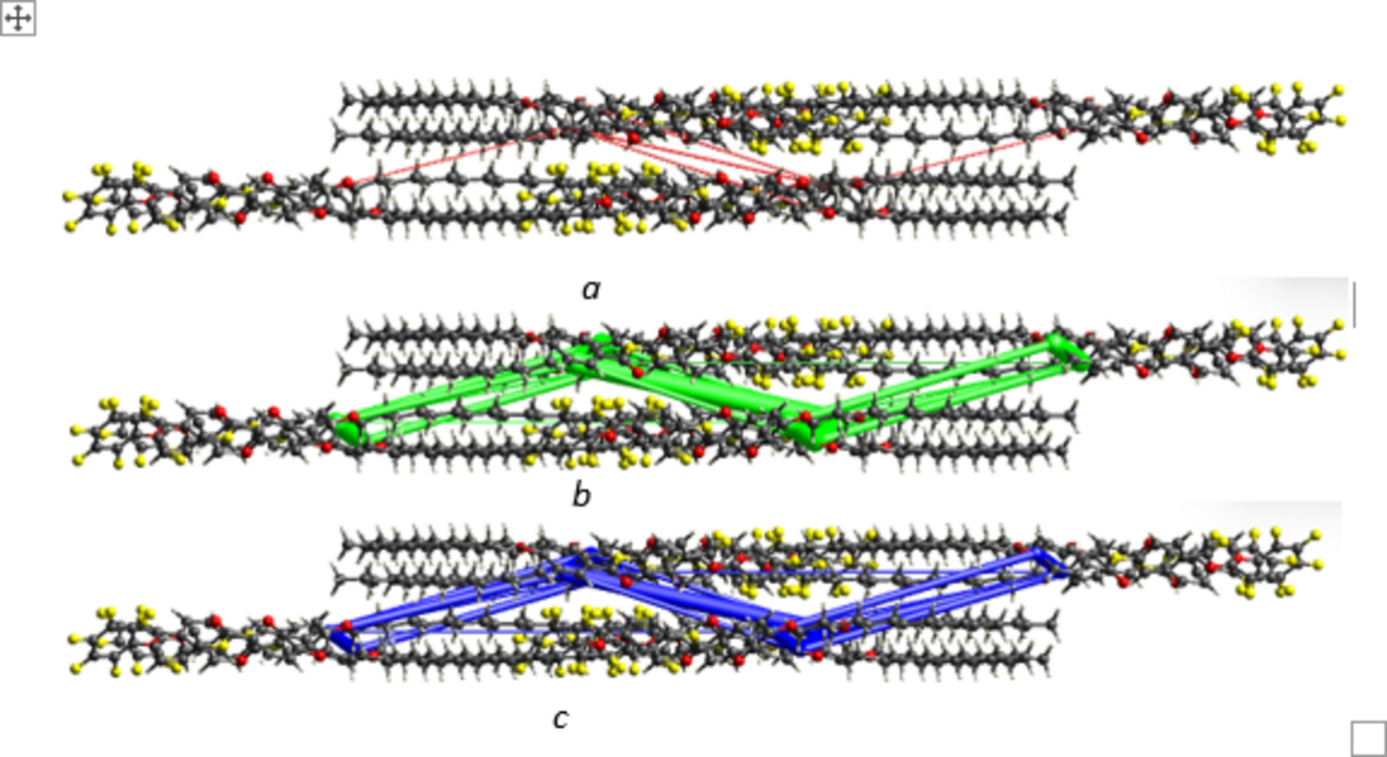
The topology of the energy frameworks for (**I**) representing Coulombic, dispersion and total energy.

The Hirshfeld surface analysis was performed using *CrystalExplorer* (Spackman *et al.*, 2021 ▸). Fig. 5 ▸ illustrates the Hirshfeld surface of (**I**) mapped over *d*_norm_ and shape-index. The red triangular-shaped region, if viewed normal to the centre of the carbonyl­phenyl ring indicates the existence of π–π stacking. The two-dimensional fingerprint plots (Fig. 6 ▸) indicate that the major contributions to the crystal packing of (**I**) are from H⋯H: (45%), F⋯H/H⋯F: (18.5%), O⋯H/H⋯O: (9.7%), C⋯H/H⋯C: (9.4%), F⋯C/C⋯F: (7.3%),, F⋯F: (1.9%) contacts. Inter­action energies for (**I**) were computed using the basis set B3LYP\631-G(d,p) for mol­ecular pairs within a cluster of 3.8 Å radius, giving *E*_ele_ = −37.6 kJ mol^−1^, *E*_pol_ = −15.6 kJ mol^−1^, *E*_dis_ = −383.4 kJ mol^−1^ and *E*_rep_ = +128.4 kJ mol^−1^. The energy framework topologies are shown in Fig. 7 ▸.

## 5. Database survey

A search of the Cambridge Structural Database (CSD version 6.01, March 2026; Groom *et al.*, 2016 ▸) for structures containing the 4-(dodec­yloxy) benzoate moiety yielded eleven hits. Among these, five structures with CSD refcodes FOCDIN (Kanji Kubo *et al.*, 2018 ▸), PUWDES, SANCAO and PUWREG (Dutronc *et al.*, 2016 ▸), and TUVCAP (Cheng *et al.*, 2010 ▸) are substituted with long alkyl chains or aromatic rings that are nearly planar, showing only slight deviations. The dihedral angles between these substituent planes and the 4-(dodec­yloxy) benzoate moiety are 82.3, 70.1, 47.5, 57.7 and 79.0°, respectively. In the title compound, the dihedral angle between the 4-(dodec­yloxy)benzoate ring and the (undecyl­oxyphen­yl)acrylate fragment is 78.98 (2)°, which lies within the range observed for related structures. However, a notable difference is observed in the torsion angle: in the reported structures, the torsion angle between the substituted oxygen atom and the adjacent atom of the almost planar fragment ranges from approximately 1° to 10°, indicating near coplanarity in those segments. In contrast, in the title compound, the torsion angle between the dodec­yloxy chain and the phenyl ring is 172.1°, indicating that these groups are nearly coplanar and adopt an anti (extended) conformation, with only a small deviation (∼8°) from the ideal 180°.

## 6. Synthesis and crystallization

The reaction mixture of 2,3,4,5,6-penta­fluoro­phenol (0.184 g, 1 eq) and 4-{[4-(dodec­yloxy)benzo­yl]­oxy}benzoic acid (0.426 g, 1 eq) in di­chloro­methane was stirred at room temperature overnight using a DCC esterification process in the presence of *N*,*N*-di­methyl­amino­pyrimidine as a catalyst. The insoluble byproduct of di­cyclo­hexyl urea was removed by filtration. The filtrate was washed with 5% acetic acid solution in water, and then with pure water. The filtrate was passed through silica gel, and then left for a week to grow crystals for X-ray studies. ^1^H NMR (500 MHz, CDCl_3_): δ 8.12–8.02 (*m*, 4H, Ar-H), 7.54 (*m*, 2H, Ar-H), 7.10 (*d*, *J* = 8.5 Hz, 2H, Ar-H), 4.01 (*t*, *J* = 6.5Hz, 2H, –OCH_2_–), 1.74–1.25 (*m*, 20H, CH_2_-alk­yl), 0.91 (*t*, *J* = 4.5Hz, 3H, –CH_3_) ppm. Elemental analysis (%) calculated: C, 64.86; H, 5.61; F, 16.03; found C, 64.90; H, 5.65; F, 16.09%.

## 7. Refinement

Crystal data, data collection and structure refinement details are summarized in Table 2 ▸. All H atoms were positioned with idealized geometry and refined using a riding model with C—H = 0.93–0.97 Å and *U*_iso_(H) = 1.2*U*_eq_(C) or 1.5*U*_eq_(methyl C).

## Supplementary Material

Crystal structure: contains datablock(s) I. DOI: 10.1107/S2056989026003920/hb8211sup1.cif

Structure factors: contains datablock(s) I. DOI: 10.1107/S2056989026003920/hb8211Isup2.hkl

Supporting information file. DOI: 10.1107/S2056989026003920/hb8211Isup3.cml

CCDC reference: 2546170

Additional supporting information:  crystallographic information; 3D view; checkCIF report

Additional supporting information:  crystallographic information; 3D view; checkCIF report

## Figures and Tables

**Table 1:** Hydrogen-bond geometry (Å, °) *Cg*2 and *Cg*3 are the centroids of the C8–C13 and C15–C20 rings, respectively.

*D*—H⋯*A*	*D*—H	H⋯*A*	*D*⋯*A*	*D*—H⋯*A*
C13—H13⋯O1	0.93	2.39	2.715 (4)	100
C16—H16⋯O3	0.93	2.42	2.730 (4)	100
C9—H9⋯O4^i^	0.93	2.60	3.250 (4)	128
C3—F2⋯*Cg*3^ii^	1.34 (1)	3.44 (1)	3.899 (5)	100 (1)
C5—F4⋯*Cg*3^iii^	1.33 (1)	3.18 (1)	3.604 (4)	98 (1)
C6—F5⋯*Cg*2^iv^	1.33 (1)	3.42 (1)	3.917 (4)	102 (1)

Symmetry codes: (i) 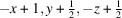; (ii) 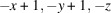; (iii) 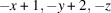; (iv) 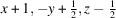.

**Table 2:** Experimental details

Crystal data	
Chemical formula	C_32_H_33_F_5_O_5_
*M*_r_	592.58
Crystal system, space group	Monoclinic, *P*2_1_/*c*
Temperature (K)	297
*a*, *b*, *c* (Å)	25.212 (3), 8.8684 (11), 13.7665 (18)
β (°)	102.518 (4)
*V* (Å^3^)	3004.8 (7)
*Z*	4
Radiation type	Mo *K*α
μ (mm^−1^)	0.11
Crystal size (mm)	0.32 × 0.27 × 0.21

Data collection	
Diffractometer	Bruker SMART APEXII CCD
Absorption correction	Multi-scan (*SADABS*; Krause *et al.*, 2015 ▸)
*T*_min_, *T*_max_	0.964, 0.976
No. of measured, independent and observed [*I* > 2σ(*I*)] reflections	64189, 6157, 3592
*R*_int_	0.108
(sin θ/λ)_max_ (Å^−1^)	0.626

Refinement	
*R*[*F*^2^ > 2σ(*F*^2^)], *wR*(*F*^2^), *S*	0.087, 0.216, 1.08
No. of reflections	6157
No. of parameters	379
H-atom treatment	H-atom parameters constrained
Δρ_max_, Δρ_min_ (e Å^−3^)	0.17, −0.20

Computer programs: *APEX2* and *SAINT* (Bruker, 2017 ▸), *SHELXT* (Sheldrick, 2015*a* ▸), *SHELXL2019/3* (Sheldrick, 2015*b* ▸), *Mercury* (Macrae *et al.*, 2020 ▸) and *publCIF* (Westrip, 2010 ▸).

## supplementary crystallographic information

### 4-[(2,3,4,5,6-pentafluorophenoxy)carbonyl]phenyl 4-(dodecyloxy)benzoate Crystal data

**Table:**

C_32_H_33_F_5_O_5_	*F*(000) = 1240
*M**_r_* = 592.58	Prism
Monoclinic, *P*2_1_/*c*	*D*_x_ = 1.310 Mg m^−^^3^
Hall symbol: -P 2ybc	Mo *K*α radiation, λ = 0.71073 Å
*a* = 25.212 (3) Å	Cell parameters from 3592 reflections
*b* = 8.8684 (11) Å	θ = 2–27°
*c* = 13.7665 (18) Å	µ = 0.11 mm^−^^1^
β = 102.518 (4)°	*T* = 297 K
*V* = 3004.8 (7) Å^3^	Prism, colourless
*Z* = 4	0.32 × 0.27 × 0.21 mm

### 4-[(2,3,4,5,6-pentafluorophenoxy)carbonyl]phenyl 4-(dodecyloxy)benzoate Data collection

**Table:**

Bruker SMART APEXII CCD diffractometer	6157 independent reflections
Radiation source: fine-focus sealed tube	3592 reflections with *I* > 2σ(*I*)
Graphite monochromator	*R*_int_ = 0.108
Detector resolution: 1.97 pixels mm^-1^	θ_max_ = 26.4°, θ_min_ = 2.8°
φ and Ω scans	*h* = −31→31
Absorption correction: multi-scan (SADABS; Krause *et al.*, 2015)	*k* = −11→10
*T*_min_ = 0.964, *T*_max_ = 0.976	*l* = −17→17
64189 measured reflections	

### 4-[(2,3,4,5,6-pentafluorophenoxy)carbonyl]phenyl 4-(dodecyloxy)benzoate Refinement

**Table:**

Refinement on *F*^2^	Primary atom site location: structure-invariant direct methods
Least-squares matrix: full	Secondary atom site location: difference Fourier map
*R*[*F*^2^ > 2σ(*F*^2^)] = 0.087	Hydrogen site location: inferred from neighbouring sites
*wR*(*F*^2^) = 0.216	H-atom parameters constrained
*S* = 1.08	*w* = 1/[σ^2^(*F*_o_^2^) + (0.0703*P*)^2^ + 1.9288*P*] where *P* = (*F*_o_^2^ + 2*F*_c_^2^)/3
6157 reflections	(Δ/σ)_max_ < 0.001
379 parameters	Δρ_max_ = 0.17 e Å^−^^3^
0 restraints	Δρ_min_ = −0.20 e Å^−^^3^
0 constraints	

### 4-[(2,3,4,5,6-pentafluorophenoxy)carbonyl]phenyl 4-(dodecyloxy)benzoate Special details

**Table:**

Geometry. All esds (except the esd in the dihedral angle between two l.s. planes) are estimated using the full covariance matrix. The cell esds are taken into account individually in the estimation of esds in distances, angles and torsion angles; correlations between esds in cell parameters are only used when they are defined by crystal symmetry. An approximate (isotropic) treatment of cell esds is used for estimating esds involving l.s. planes.

### 4-[(2,3,4,5,6-pentafluorophenoxy)carbonyl]phenyl 4-(dodecyloxy)benzoate Fractional atomic coordinates and isotropic or equivalent isotropic displacement parameters (Å^2^)

**Table:**

	*x*	*y*	*z*	*U*_iso_*/*U*_eq_	
O2	0.62659 (10)	0.8834 (3)	−0.00610 (18)	0.0711 (7)	
O3	0.39899 (9)	0.7824 (3)	0.11380 (17)	0.0692 (7)	
O1	0.58408 (10)	0.7223 (3)	−0.12246 (18)	0.0797 (8)	
O4	0.43103 (11)	0.6248 (3)	0.2388 (2)	0.0893 (9)	
O5	0.20585 (10)	0.8468 (3)	0.32820 (19)	0.0843 (8)	
C1	0.62584 (15)	0.7373 (4)	−0.1729 (3)	0.0635 (9)	
C2	0.67148 (17)	0.6497 (5)	−0.1492 (3)	0.0745 (11)	
C3	0.71051 (16)	0.6580 (5)	−0.2045 (3)	0.0820 (12)	
C4	0.70352 (17)	0.7543 (5)	−0.2838 (3)	0.0784 (12)	
C5	0.65836 (16)	0.8418 (4)	−0.3073 (3)	0.0676 (10)	
C6	0.61994 (14)	0.8345 (4)	−0.2515 (3)	0.0628 (9)	
C7	0.58815 (15)	0.8070 (4)	−0.0374 (2)	0.0542 (8)	
C8	0.53892 (13)	0.7883 (3)	0.0036 (2)	0.0502 (8)	
C9	0.53588 (13)	0.8741 (4)	0.0864 (2)	0.0536 (8)	
H9	0.564725	0.936244	0.115161	0.064*	
C10	0.49039 (14)	0.8676 (4)	0.1260 (2)	0.0573 (9)	
H10	0.488360	0.923881	0.182060	0.069*	
C11	0.44813 (14)	0.7770 (4)	0.0817 (2)	0.0581 (9)	
C12	0.45047 (15)	0.6890 (4)	0.0004 (3)	0.0678 (10)	
H12	0.421761	0.625661	−0.027261	0.081*	
C13	0.49620 (15)	0.6963 (4)	−0.0393 (3)	0.0649 (9)	
H13	0.498183	0.639122	−0.095040	0.078*	
C14	0.39587 (14)	0.7057 (4)	0.1983 (3)	0.0598 (9)	
C15	0.34493 (13)	0.7394 (4)	0.2292 (2)	0.0576 (8)	
C16	0.30507 (14)	0.8343 (4)	0.1765 (3)	0.0622 (9)	
H16	0.309606	0.877081	0.117177	0.075*	
C17	0.25925 (14)	0.8656 (4)	0.2107 (3)	0.0692 (10)	
H17	0.232581	0.927649	0.173849	0.083*	
C18	0.25225 (14)	0.8051 (4)	0.3004 (3)	0.0640 (9)	
C19	0.29130 (15)	0.7095 (5)	0.3536 (3)	0.0752 (11)	
H19	0.286706	0.666733	0.412878	0.090*	
C20	0.33676 (15)	0.6785 (4)	0.3181 (3)	0.0713 (10)	
H20	0.363031	0.614770	0.354462	0.086*	
C21	0.20080 (16)	0.8107 (5)	0.4269 (3)	0.0887 (13)	
H21A	0.230112	0.856987	0.474917	0.106*	
H21B	0.202828	0.702320	0.436681	0.106*	
C22	0.14732 (15)	0.8682 (6)	0.4411 (3)	0.0866 (13)	
H22A	0.118463	0.819903	0.393057	0.104*	
H22B	0.145254	0.975724	0.427945	0.104*	
C23	0.13827 (16)	0.8402 (6)	0.5441 (3)	0.0934 (14)	
H23A	0.145117	0.734372	0.559865	0.112*	
H23B	0.164865	0.898047	0.590906	0.112*	
C24	0.08310 (16)	0.8786 (6)	0.5598 (3)	0.0916 (13)	
H24A	0.075818	0.983454	0.541695	0.110*	
H24B	0.056675	0.818229	0.514387	0.110*	
C25	0.07406 (17)	0.8562 (6)	0.6622 (3)	0.0967 (14)	
H25A	0.085332	0.754546	0.683199	0.116*	
H25B	0.097722	0.924978	0.706441	0.116*	
C26	0.01770 (18)	0.8783 (6)	0.6763 (3)	0.1067 (16)	
H26A	0.006387	0.979361	0.653921	0.128*	
H26B	−0.005702	0.808603	0.632363	0.128*	
C27	0.00738 (18)	0.8593 (6)	0.7763 (3)	0.1045 (16)	
H27A	0.029293	0.932870	0.819433	0.125*	
H27B	0.020558	0.760344	0.800135	0.125*	
C28	−0.04948 (18)	0.8736 (7)	0.7896 (3)	0.1110 (17)	
H28A	−0.062671	0.972398	0.765482	0.133*	
H28B	−0.071345	0.799824	0.746527	0.133*	
C29	−0.06010 (19)	0.8552 (7)	0.8893 (4)	0.1158 (18)	
H29A	−0.045840	0.757473	0.913831	0.139*	
H29B	−0.038712	0.930481	0.931699	0.139*	
C30	−0.1156 (2)	0.8649 (7)	0.9048 (4)	0.1215 (19)	
H30A	−0.130530	0.960962	0.878194	0.146*	
H30B	−0.136808	0.786771	0.864814	0.146*	
C31	−0.1246 (3)	0.8517 (8)	1.0055 (5)	0.152 (3)	
H31A	−0.108028	0.757934	1.032921	0.183*	
H31B	−0.104544	0.932616	1.044445	0.183*	
C32	−0.1787 (3)	0.8546 (9)	1.0231 (5)	0.175 (3)	
H32A	−0.177098	0.844603	1.093133	0.263*	
H32B	−0.195806	0.948374	0.999799	0.263*	
H32C	−0.199313	0.772584	0.988201	0.263*	
F5	0.57632 (9)	0.9232 (3)	−0.27505 (17)	0.0880 (7)	
F4	0.65212 (11)	0.9356 (3)	−0.38455 (17)	0.0974 (8)	
F3	0.74166 (10)	0.7621 (3)	−0.3378 (2)	0.1192 (10)	
F1	0.67773 (12)	0.5529 (3)	−0.07250 (18)	0.1108 (9)	
F2	0.75492 (11)	0.5723 (4)	−0.1801 (2)	0.1260 (10)	

### 4-[(2,3,4,5,6-pentafluorophenoxy)carbonyl]phenyl 4-(dodecyloxy)benzoate Atomic displacement parameters (Å^2^)

**Table:**

	*U*^11^	*U*^22^	*U*^33^	*U*^12^	*U*^13^	*U*^23^
O2	0.0704 (16)	0.0849 (18)	0.0659 (16)	−0.0158 (14)	0.0323 (13)	−0.0230 (14)
O3	0.0610 (14)	0.0841 (17)	0.0701 (16)	0.0127 (12)	0.0309 (12)	0.0216 (13)
O1	0.0848 (18)	0.101 (2)	0.0663 (16)	−0.0254 (15)	0.0441 (14)	−0.0310 (15)
O4	0.0836 (19)	0.105 (2)	0.092 (2)	0.0353 (17)	0.0459 (16)	0.0468 (17)
O5	0.0609 (15)	0.122 (2)	0.0768 (18)	0.0116 (15)	0.0292 (13)	0.0103 (16)
C1	0.068 (2)	0.075 (2)	0.054 (2)	−0.0108 (19)	0.0287 (18)	−0.0192 (19)
C2	0.087 (3)	0.085 (3)	0.057 (2)	0.004 (2)	0.027 (2)	−0.003 (2)
C3	0.066 (2)	0.097 (3)	0.084 (3)	0.021 (2)	0.017 (2)	−0.013 (2)
C4	0.076 (3)	0.101 (3)	0.072 (3)	−0.005 (2)	0.045 (2)	−0.017 (2)
C5	0.085 (3)	0.072 (2)	0.052 (2)	−0.005 (2)	0.029 (2)	−0.0076 (19)
C6	0.063 (2)	0.070 (2)	0.058 (2)	0.0012 (19)	0.0206 (18)	−0.0172 (19)
C7	0.073 (2)	0.0474 (18)	0.0469 (19)	0.0040 (17)	0.0237 (17)	0.0001 (15)
C8	0.063 (2)	0.0490 (18)	0.0421 (17)	0.0023 (16)	0.0202 (15)	0.0013 (14)
C9	0.061 (2)	0.056 (2)	0.0489 (18)	−0.0051 (15)	0.0213 (15)	−0.0039 (15)
C10	0.067 (2)	0.063 (2)	0.0477 (19)	0.0049 (18)	0.0237 (17)	−0.0023 (16)
C11	0.061 (2)	0.063 (2)	0.055 (2)	0.0074 (18)	0.0221 (17)	0.0130 (17)
C12	0.065 (2)	0.072 (2)	0.071 (2)	−0.0114 (18)	0.0229 (19)	−0.008 (2)
C13	0.075 (2)	0.066 (2)	0.057 (2)	−0.0038 (19)	0.0227 (19)	−0.0104 (18)
C14	0.064 (2)	0.059 (2)	0.060 (2)	0.0009 (18)	0.0204 (18)	0.0140 (18)
C15	0.058 (2)	0.058 (2)	0.060 (2)	0.0003 (16)	0.0205 (17)	0.0056 (17)
C16	0.062 (2)	0.073 (2)	0.053 (2)	−0.0039 (18)	0.0164 (17)	0.0042 (18)
C17	0.054 (2)	0.089 (3)	0.065 (2)	0.0060 (19)	0.0140 (18)	0.009 (2)
C18	0.056 (2)	0.076 (2)	0.064 (2)	−0.0037 (19)	0.0223 (18)	−0.0016 (19)
C19	0.073 (3)	0.090 (3)	0.072 (2)	0.010 (2)	0.035 (2)	0.018 (2)
C20	0.074 (2)	0.077 (3)	0.070 (2)	0.013 (2)	0.031 (2)	0.017 (2)
C21	0.076 (3)	0.115 (4)	0.085 (3)	0.003 (2)	0.039 (2)	0.006 (3)
C22	0.063 (2)	0.120 (4)	0.083 (3)	−0.001 (2)	0.030 (2)	−0.004 (3)
C23	0.070 (3)	0.131 (4)	0.087 (3)	0.004 (3)	0.034 (2)	0.003 (3)
C24	0.072 (3)	0.123 (4)	0.088 (3)	0.009 (3)	0.034 (2)	0.005 (3)
C25	0.077 (3)	0.138 (4)	0.083 (3)	0.007 (3)	0.034 (2)	0.008 (3)
C26	0.083 (3)	0.156 (5)	0.092 (3)	0.021 (3)	0.044 (3)	0.021 (3)
C27	0.082 (3)	0.150 (5)	0.090 (3)	0.017 (3)	0.038 (3)	0.017 (3)
C28	0.090 (3)	0.155 (5)	0.100 (4)	0.030 (3)	0.048 (3)	0.026 (3)
C29	0.096 (3)	0.162 (5)	0.103 (4)	0.025 (3)	0.050 (3)	0.031 (3)
C30	0.096 (3)	0.160 (5)	0.127 (4)	0.029 (3)	0.065 (3)	0.027 (4)
C31	0.143 (5)	0.195 (7)	0.146 (5)	0.024 (5)	0.092 (4)	0.032 (5)
C32	0.164 (6)	0.211 (7)	0.187 (7)	0.032 (6)	0.121 (5)	0.050 (6)
F5	0.0857 (16)	0.0905 (16)	0.0893 (16)	0.0190 (13)	0.0221 (12)	−0.0165 (13)
F4	0.132 (2)	0.0965 (17)	0.0726 (15)	−0.0045 (15)	0.0419 (14)	0.0069 (13)
F3	0.1030 (19)	0.162 (3)	0.119 (2)	−0.0048 (18)	0.0805 (17)	−0.0161 (19)
F1	0.144 (2)	0.119 (2)	0.0723 (16)	0.0181 (17)	0.0282 (15)	0.0150 (15)
F2	0.0914 (19)	0.154 (3)	0.132 (2)	0.0431 (18)	0.0236 (17)	−0.011 (2)

### 4-[(2,3,4,5,6-pentafluorophenoxy)carbonyl]phenyl 4-(dodecyloxy)benzoate Geometric parameters (Å, º)

**Table:**

O2—C7	1.184 (4)	C19—H19	0.9300
O3—C14	1.365 (4)	C20—H20	0.9300
O3—C11	1.404 (4)	C21—C22	1.494 (5)
O1—C7	1.376 (4)	C21—H21A	0.9700
O1—C1	1.387 (4)	C21—H21B	0.9700
O4—C14	1.182 (4)	C22—C23	1.505 (5)
O5—C18	1.358 (4)	C22—H22A	0.9700
O5—C21	1.428 (4)	C22—H22B	0.9700
C1—C6	1.366 (5)	C23—C24	1.493 (5)
C1—C2	1.368 (5)	C23—H23A	0.9700
C2—F1	1.344 (4)	C23—H23B	0.9700
C2—C3	1.370 (5)	C24—C25	1.490 (5)
C3—F2	1.335 (4)	C24—H24A	0.9700
C3—C4	1.368 (6)	C24—H24B	0.9700
C4—F3	1.338 (4)	C25—C26	1.488 (5)
C4—C5	1.358 (5)	C25—H25A	0.9700
C5—F4	1.333 (4)	C25—H25B	0.9700
C5—C6	1.362 (5)	C26—C27	1.466 (6)
C6—F5	1.334 (4)	C26—H26A	0.9700
C7—C8	1.481 (4)	C26—H26B	0.9700
C8—C13	1.378 (5)	C27—C28	1.489 (5)
C8—C9	1.386 (4)	C27—H27A	0.9700
C9—C10	1.374 (4)	C27—H27B	0.9700
C9—H9	0.9300	C28—C29	1.464 (6)
C10—C11	1.368 (5)	C28—H28A	0.9700
C10—H10	0.9300	C28—H28B	0.9700
C11—C12	1.376 (5)	C29—C30	1.463 (6)
C12—C13	1.380 (5)	C29—H29A	0.9700
C12—H12	0.9300	C29—H29B	0.9700
C13—H13	0.9300	C30—C31	1.458 (7)
C14—C15	1.469 (5)	C30—H30A	0.9700
C15—C16	1.389 (5)	C30—H30B	0.9700
C15—C20	1.393 (4)	C31—C32	1.435 (7)
C16—C17	1.367 (5)	C31—H31A	0.9700
C16—H16	0.9300	C31—H31B	0.9700
C17—C18	1.392 (5)	C32—H32A	0.9600
C17—H17	0.9300	C32—H32B	0.9600
C18—C19	1.382 (5)	C32—H32C	0.9600
C19—C20	1.367 (5)		
			
C14—O3—C11	118.0 (3)	O5—C21—H21A	110.0
C7—O1—C1	116.6 (3)	C22—C21—H21A	110.0
C18—O5—C21	117.8 (3)	O5—C21—H21B	110.0
C6—C1—C2	119.2 (3)	C22—C21—H21B	110.0
C6—C1—O1	119.5 (3)	H21A—C21—H21B	108.4
C2—C1—O1	121.1 (4)	C21—C22—C23	113.1 (4)
C6—C1—O1	119.5 (3)	C21—C22—H22A	109.0
C2—C1—O1	121.1 (4)	C23—C22—H22A	109.0
F1—C2—C1	119.9 (3)	C21—C22—H22B	109.0
F1—C2—C3	119.6 (4)	C23—C22—H22B	109.0
C1—C2—C3	120.4 (4)	H22A—C22—H22B	107.8
F2—C3—C4	120.8 (4)	C24—C23—C22	115.8 (4)
F2—C3—C2	119.6 (4)	C24—C23—H23A	108.3
C4—C3—C2	119.5 (4)	C22—C23—H23A	108.3
F3—C4—C5	120.4 (4)	C24—C23—H23B	108.3
F3—C4—C3	119.5 (4)	C22—C23—H23B	108.3
C5—C4—C3	120.2 (3)	H23A—C23—H23B	107.4
F4—C5—C4	119.6 (3)	C25—C24—C23	116.4 (4)
F4—C5—C6	120.3 (4)	C25—C24—H24A	108.2
C4—C5—C6	120.1 (4)	C23—C24—H24A	108.2
F5—C6—C5	119.0 (4)	C25—C24—H24B	108.2
F5—C6—C1	120.5 (3)	C23—C24—H24B	108.2
C5—C6—C1	120.6 (4)	H24A—C24—H24B	107.3
O2—C7—O1	121.8 (3)	C26—C25—C24	116.8 (4)
O2—C7—O1	121.8 (3)	C26—C25—H25A	108.1
O2—C7—C8	127.8 (3)	C24—C25—H25A	108.1
O1—C7—C8	110.4 (3)	C26—C25—H25B	108.1
O1—C7—C8	110.4 (3)	C24—C25—H25B	108.1
C13—C8—C9	120.0 (3)	H25A—C25—H25B	107.3
C13—C8—C7	123.0 (3)	C27—C26—C25	118.4 (4)
C9—C8—C7	116.9 (3)	C27—C26—H26A	107.7
C10—C9—C8	120.2 (3)	C25—C26—H26A	107.7
C10—C9—H9	119.9	C27—C26—H26B	107.7
C8—C9—H9	119.9	C25—C26—H26B	107.7
C11—C10—C9	119.0 (3)	H26A—C26—H26B	107.1
C11—C10—H10	120.5	C26—C27—C28	118.5 (4)
C9—C10—H10	120.5	C26—C27—H27A	107.7
C10—C11—C12	122.0 (3)	C28—C27—H27A	107.7
C10—C11—O3	119.7 (3)	C26—C27—H27B	107.7
C12—C11—O3	118.1 (3)	C28—C27—H27B	107.7
C10—C11—O3	119.7 (3)	H27A—C27—H27B	107.1
C12—C11—O3	118.1 (3)	C29—C28—C27	118.8 (4)
C11—C12—C13	118.8 (3)	C29—C28—H28A	107.6
C11—C12—H12	120.6	C27—C28—H28A	107.6
C13—C12—H12	120.6	C29—C28—H28B	107.6
C8—C13—C12	120.0 (3)	C27—C28—H28B	107.6
C8—C13—H13	120.0	H28A—C28—H28B	107.0
C12—C13—H13	120.0	C30—C29—C28	120.3 (4)
O4—C14—O3	121.9 (3)	C30—C29—H29A	107.2
O4—C14—O3	121.9 (3)	C28—C29—H29A	107.2
O4—C14—C15	126.9 (3)	C30—C29—H29B	107.2
O3—C14—C15	111.2 (3)	C28—C29—H29B	107.2
O3—C14—C15	111.2 (3)	H29A—C29—H29B	106.9
C16—C15—C20	117.8 (3)	C31—C30—C29	119.0 (5)
C16—C15—C14	123.4 (3)	C31—C30—H30A	107.6
C20—C15—C14	118.7 (3)	C29—C30—H30A	107.6
C17—C16—C15	120.8 (3)	C31—C30—H30B	107.6
C17—C16—H16	119.6	C29—C30—H30B	107.6
C15—C16—H16	119.6	H30A—C30—H30B	107.0
C16—C17—C18	120.4 (3)	C32—C31—C30	120.6 (6)
C16—C17—H17	119.8	C32—C31—H31A	107.2
C18—C17—H17	119.8	C30—C31—H31A	107.2
O5—C18—C19	125.2 (3)	C32—C31—H31B	107.2
O5—C18—C17	115.3 (3)	C30—C31—H31B	107.2
C19—C18—C17	119.5 (3)	H31A—C31—H31B	106.8
C20—C19—C18	119.4 (3)	C31—C32—H32A	109.5
C20—C19—H19	120.3	C31—C32—H32B	109.5
C18—C19—H19	120.3	H32A—C32—H32B	109.5
C19—C20—C15	122.0 (4)	C31—C32—H32C	109.5
C19—C20—H20	119.0	H32A—C32—H32C	109.5
C15—C20—H20	119.0	H32B—C32—H32C	109.5
O5—C21—C22	108.5 (3)		
			
C7—O1—C1—C6	−96.2 (4)	C9—C10—C11—O3	−172.5 (3)
C7—O1—C1—C2	87.9 (4)	C9—C10—C11—O3	−172.5 (3)
C6—C1—C2—F1	−179.6 (3)	O3—O3—C11—C10	0.00 (12)
O1—C1—C2—F1	−3.6 (5)	C14—O3—C11—C10	−79.7 (4)
O1—C1—C2—F1	−3.6 (5)	C14—O3—C11—C12	105.6 (4)
C6—C1—C2—C3	−0.8 (6)	C10—C11—C12—C13	−2.1 (5)
O1—C1—C2—C3	175.1 (3)	O3—C11—C12—C13	172.4 (3)
O1—C1—C2—C3	175.1 (3)	O3—C11—C12—C13	172.4 (3)
F1—C2—C3—F2	−1.6 (6)	C9—C8—C13—C12	−0.3 (5)
C1—C2—C3—F2	179.7 (4)	C7—C8—C13—C12	−177.2 (3)
F1—C2—C3—C4	178.6 (4)	C11—C12—C13—C8	1.3 (5)
C1—C2—C3—C4	−0.1 (6)	C11—O3—C14—O4	−7.3 (5)
F2—C3—C4—F3	0.2 (6)	C11—O3—C14—O3	0 (56)
C2—C3—C4—F3	−179.9 (4)	C11—O3—C14—C15	172.2 (3)
F2—C3—C4—C5	−179.4 (4)	O4—C14—C15—C16	−178.9 (4)
C2—C3—C4—C5	0.4 (6)	O3—C14—C15—C16	1.6 (5)
F3—C4—C5—F4	0.1 (6)	O3—C14—C15—C16	1.6 (5)
C3—C4—C5—F4	179.7 (4)	O4—C14—C15—C20	3.8 (6)
F3—C4—C5—C6	−179.3 (3)	O3—C14—C15—C20	−175.6 (3)
C3—C4—C5—C6	0.3 (6)	O3—C14—C15—C20	−175.6 (3)
F4—C5—C6—F5	−0.4 (5)	C20—C15—C16—C17	−0.5 (5)
C4—C5—C6—F5	179.0 (3)	C14—C15—C16—C17	−177.8 (3)
F4—C5—C6—C1	179.3 (3)	C15—C16—C17—C18	1.3 (6)
C4—C5—C6—C1	−1.3 (6)	C21—O5—C18—C19	11.1 (6)
C2—C1—C6—F5	−178.8 (3)	C21—O5—C18—C17	−169.2 (3)
O1—C1—C6—F5	5.2 (5)	C16—C17—C18—O5	178.5 (3)
O1—C1—C6—F5	5.2 (5)	C16—C17—C18—C19	−1.8 (6)
C2—C1—C6—C5	1.6 (5)	O5—C18—C19—C20	−178.9 (4)
O1—C1—C6—C5	−174.4 (3)	C17—C18—C19—C20	1.4 (6)
O1—C1—C6—C5	−174.4 (3)	C18—C19—C20—C15	−0.5 (6)
C1—O1—C7—O2	−3.6 (5)	C16—C15—C20—C19	0.1 (6)
C1—O1—C7—O1	0 (82)	C14—C15—C20—C19	177.5 (4)
C1—O1—C7—C8	175.6 (3)	C18—O5—C21—C22	179.4 (3)
O2—C7—C8—C13	179.8 (3)	O5—C21—C22—C23	−178.3 (4)
O1—C7—C8—C13	0.6 (5)	C21—C22—C23—C24	−173.1 (4)
O1—C7—C8—C13	0.6 (5)	C22—C23—C24—C25	−178.1 (4)
O2—C7—C8—C9	2.8 (5)	C23—C24—C25—C26	−173.6 (5)
O1—C7—C8—C9	−176.4 (3)	C24—C25—C26—C27	−179.2 (5)
O1—C7—C8—C9	−176.4 (3)	C25—C26—C27—C28	−176.9 (5)
C13—C8—C9—C10	0.1 (5)	C26—C27—C28—C29	−179.8 (5)
C7—C8—C9—C10	177.2 (3)	C27—C28—C29—C30	−178.6 (5)
C8—C9—C10—C11	−0.9 (5)	C28—C29—C30—C31	−177.7 (6)
C9—C10—C11—C12	1.9 (5)	C29—C30—C31—C32	−177.4 (6)

### 4-[(2,3,4,5,6-pentafluorophenoxy)carbonyl]phenyl 4-(dodecyloxy)benzoate Hydrogen-bond geometry (Å, º)

*Cg*2 and *Cg*3 are the centroids of the C8–C13 and C15–C20 rings, respectively.

**Table:**

*D*—H···*A*	*D*—H	H···*A*	*D*···*A*	*D*—H···*A*
C13—H13···O1	0.93	2.39	2.715 (4)	100
C16—H16···O3	0.93	2.42	2.730 (4)	100
C9—H9···O4^i^	0.93	2.60	3.250 (4)	128
C3—F2···*Cg*3^ii^	1.34 (1)	3.44 (1)	3.899 (5)	100 (1)
C5—F4···*Cg*3^iii^	1.33 (1)	3.18 (1)	3.604 (4)	98 (1)
C6—F5···*Cg*2^iv^	1.33 (1)	3.42 (1)	3.917 (4)	102 (1)

Symmetry codes: (i) −*x*+1, *y*+1/2, −*z*+1/2; (ii) −*x*+1, −*y*+1, −*z*; (iii) −*x*+1, −*y*+2, −*z*; (iv) *x*+1, −*y*+1/2, *z*−1/2.
